# Development of Clinical Practice Guidelines and Primary Care Referral Pathways for the Management of Otorhinolaryngological Conditions in Pakistan

**DOI:** 10.1055/s-0046-1818583

**Published:** 2026-04-24

**Authors:** Alina Pervez, Russell Seth Martins, Huzaifa Moiz, Abbas Raza Syed, Muneeb Khan, Nashia Ali Rizvi, Mohsin Ali Mustafa, Muhammad Taha Nasim, Alina Abdul Rehman, Shayan Khalid, Saif Ur Rehman, Sarah Nadeem, Adil H. Haider, Shabbir Akhtar

**Affiliations:** 1 Center for Clinical Best Practices (CCBP), Clinical and Translational Research Incubator (CITRIC), Medical College, Aga Khan University, Karachi, Sindh, Pakistan; 2 Section of Otolaryngology, Head and Neck Surgery, Department of Surgery, Medical College, Aga Khan University, Karachi, Sindh, Pakistan; 3 Medical College, Aga Khan University, Karachi, Sindh, Pakistan

**Keywords:** otolaryngology, referral and consultation, GRADE approach, Pakistan, clinical practice guideline

## Abstract

**Introduction:**

Developing region-specific clinical practice guidelines (CPGs) for ear, nose, and throat (ENT) diseases is crucial in Pakistan's primary care, given their significant contribution to clinical cases, aiming to enhance healthcare standards through evidence-based practices with local adaptations.

**Objectives:**

To ensure the standardization of primary healthcare and reduce unnecessary specialist referrals by creating CPGs that are appropriate to our region.

**Methods:**

We selected eight guidelines regarding epistaxis, neck masses, hearing loss, Ménière's disease, dysphonia, allergic rhinitis, acute otitis externa, and rhinosinusitis from the American Academy of Otolaryngology–Head and Neck Surgery Foundation as the source guidelines, and employed the Grading of Recommendations, Assessment, Development, and Evaluation–Adoption, Adaptation, and De Novo Development (GRADE-ADOLOPMENT) approach to contextualize guidelines by adopting, adapting, or excluding recommendations from them. Clinical-referral algorithms were created using recommendations from the CPGs created, with additional recommendations sought through a best-evidence review process.

**Results:**

We developed local CPGs for eight ENT conditions using the GRADE-ADOLOPMENT approach. While most recommendations were adopted in the local CPGs, one recommendation for acute otitis externa, hearing loss, and epistaxis and two for allergic rhinitis were adopted with minor changes. Six recommendations were excluded due to service limitations in Pakistan. Additionally, we created 8 clinical-referral algorithms, incorporating 17 additional recommendations to address gaps in practice, distributed across various conditions.

**Conclusion:**

The newly-established CPGs are instrumental in delivering standardized, high-quality care at the primary care level. Simultaneously, the development of clinical referral pathways empowers general physicians to manage patients effectively and make timely, appropriate referrals to ENT specialists.

## Introduction

Diseases of the ear, nose, and throat (ENT) account for a significant portion of the practice of a primary care physician. These diseases affect populations of all ages and are a serious concern for public health worldwide. 1 The most common ENT disorders with which patients present to an outpatient setting include otitis media and externa, cerumen impaction, hearing loss, epistaxis, allergic rhinitis, sinusitis, and pharyngitis/tonsillitis. 2 3 Several low- and middle-income countries (LMICs) have a scarcity of ENT specialists and inadequate facilities to support them, resulting in a heavy burden on the existing workforce. 4 In Pakistan, ∼ 25% of the consultations of a general physician (GP) with adults and 40% of those with children involve ENT-related complaints. 5

Improving the quality and performance of healthcare services is a priority for healthcare settings. Evidence-based clinical practice guidelines (CPGs) are the gold standard to diagnose and manage diseases, and they lead to improved patient safety and outcomes. 6 Several CPGs for ENT disorders used on an international level have been developed by high-income countries (HICs) such as the United States (US) and the United Kingdom (UK), 7 8 and are tailored to fit their healthcare systems. However, more often than not, LMICs are deficient in monetary resources and the necessary research infrastructure required to generate evidence-based CPGs on their own. 9 Pakistan, a LIMC, faces a shortage of specialist doctors, which is expected to increase further by 2030. 10 As a result, GPs oversee care provision for a vast burden of ENT conditions. A survey conducted in 2016 5 showed that most GPs in Pakistan received unsatisfactory ENT training during house job/foundation training. Thus, there is a need for the development of comprehensive CPGs for the local context in Pakistan, so that GPs can adequately provide standardized care, reduce the load of hospital care, prevent needless ENT referrals, and enhance primary health care. 11

Creating CPGs from scratch is an arduous process. It is often not possible due to inadequate resources, in which case the process should depend on a combination of adoption (integrating current recommendations as they are), adaptation (revising specific recommendations according to local context), and de novo development of recommendations, 9 a process that has been termed *adolopment* . The Grading of Recommendations, Assessment, Development, and Evaluation–Adoption, Adaptation, and De Novo Development (GRADE-ADOLOPMENT) approach uses evidence-to-decision (EtD) tables to dictate the process of adaptation. 12 13 14 These tables deliver general and context-specific evidence across fixed criteria against which experts make decisions regarding the validity of current recommendations and suggested modifications. The GRADE-ADOLOPMENT approach has been used to create CPGs in many countries, such as Saudi Arabia, 9 Tunisia, 15 Mexico, 16 countries in the Eastern Mediterranean region, 17 and Australia. 18 In Pakistan, this process has also been used to create local CPGs for the management of adult type-2 diabetes mellitus. 19

As the burden of ENT-related diseases continues to rise in Pakistan, the existing GP workforce is becoming increasingly overwhelmed. 20 To achieve optimal standards of health care, it is essential to formulate CPGs through a clear and standardized process that uses current evidence-based CPGs with relevant region-specific alterations. These local CPGs would improve ENT-related healthcare delivery in Pakistan and would have high reliability due to the transparent development processes. Primary care clinical pathways that guide primary care management and appropriate specialist referral can help streamline ENT-related primary care and reduce unnecessary specialist referrals. In the present study, we describe our use of the GRADE-ADOLOPMENT approach to develop local evidence-based CPGs and primary care clinical-referral algorithms for the management of ENT conditions at the primary care level in Pakistan.

## Methods

### Study Setting

The current study was conducted at the [name deleted to maintain the integrity of the review process], a center created in the [name deleted to maintain the integrity of the review process] at [name deleted to maintain the integrity of the review process], Karachi, Pakistan. [name deleted to maintain the integrity of the review process] is one of Pakistan's leading healthcare and biomedical research facilities.

The [name deleted to maintain the integrity of the review process] is tasked with the development of CPGs and primary care management and referral pathways to standardize the clinical practice across Pakistan. The [name deleted to maintain the integrity of the review process] collaborated with the Section of ENT at [name deleted to maintain the integrity of the review process] and the US GRADE working group to implement the GRADE-ADOLOPMENT approach to develop CPGs for eight ENT-related complaints that are commonly presented to a primary care practitioner. The primary intended audience for these CPGs includes the GPs of Pakistan.

### Study Team

The [name deleted to maintain the integrity of the review process] research team and experts from the [name deleted to maintain the integrity of the review process] Section of ENT formed the study team. The [name deleted to maintain the integrity of the review process] team members received extensive training in the GRADE-ADOLOPMENT approach and in the creation of CPGs.

### Topic Selection

The [name deleted to maintain the integrity of the review process] team approached the Section of ENT to identify the most common ENT disorders based on their clinical experience in Pakistan. The faculty selected eight topics, namely epistaxis, neck masses, sudden sensorineural hearing loss (SSNHL), Ménière's disease, dysphonia, allergic rhinitis (AR), rhinosinusitis, and acute otitis externa (AOE).

### Selection of Source Guideline

After the selection of topics, the process for the selection of source guidelines was initiated. The source guideline is the original standardized CPG that is chosen to undergo the GRADE-ADOLOPMENT approach for the development of the local guideline. Two ENT specialists appraised several source CPGs after conducting an extensive literature search on MEDLINE and Google Scholar from 2010 to September 2021. For each CPG, characteristics such as scope, local familiarity and application, rigor, and legitimacy of the establishing bodies were taken into account. As a result, the following source guidelines were selected for the creation of local CPGs:

“Clinical Practice Guideline: Acute Otitis Externa” – American Academy of Otolaryngology-Head and Neck Surgery Foundation, 2014. 21
“Clinical Practice Guideline: Hoarseness (Dysphonia) (Update)” – American Academy of Otolaryngology—Head and Neck Surgery Foundation, 2018. 22
“Clinical Practice Guideline: Nosebleed (Epistaxis) Executive Summary” –American Academy of Otolaryngology–Head, and Neck Surgery Foundation, 2020. 23
“Clinical Practice Guideline: Allergic Rhinitis” – American Academy of Otolaryngology–Head, and Neck Surgery Foundation, 2015. 24
“Clinical Practice Guideline: Evaluation of the Neck Mass in Adults” – American Academy of Otolaryngology–Head, and Neck Surgery Foundation, 2017. 25
“Clinical Practice Guideline: Sudden Hearing Loss (Update)” – American Academy of Otolaryngology–Head, and Neck Surgery Foundation, 2019. 26
“Clinical Practice Guideline (Update): Adult Sinusitis” – American Academy of Otolaryngology–Head, and Neck Surgery Foundation, 2015. 27
“Clinical Practice Guideline: Ménière's Disease” – American Academy of Otolaryngology–Head, and Neck Surgery Foundation, 2020. 7

### Review of Source Guideline

**Fig. 1:**
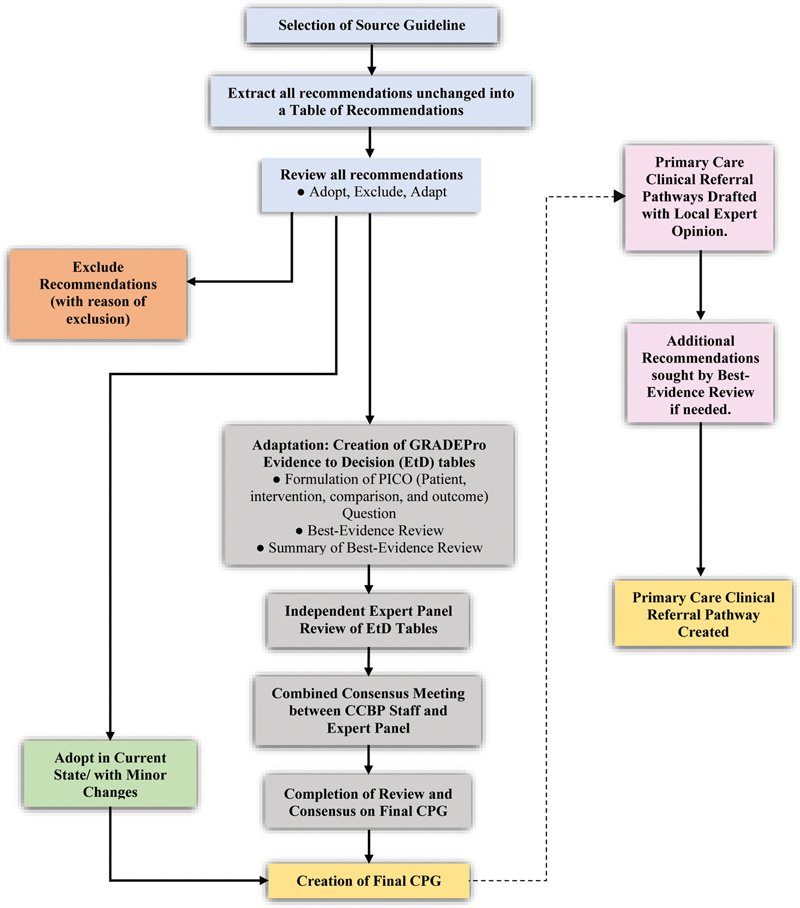
Outline of the Grading of Recommendations, Assessment, Development, and Evaluation–Adoption, Adaptation, and De Novo Development (GRADE-ADOLOPMENT) approach.

The [name deleted to maintain the integrity of the review process] team collaborated with the US GRADE working group to form an adaptation of the GRADE-ADOLOPMENT approach, and the steps are outlined in Fig. 1 . The modified GRADE-ADOLOPMENT approach has been used previously by the [name deleted to maintain the integrity of the review process] team to create guidelines for type-2 diabetes mellitus. 19 A detailed description of the steps of our modified adolopment process can be found in **Additional File 1** .

Our modified adolopment process has one important difference from the original process: recommendations that required straightforward changes which did not modify the essence of the recommendation but only provided auxiliary information were not put through the complete adaptation process (entailing EtD tables and expert panel review). In our modified adolopment process, adaptation via EtD tables and expert panel reviews is only performed if a content change is deemed necessary in a specific recommendation.

### Focus Group Discussion to Identify Challenges and Solutions

Two focus group discussions (FGDs) were held to identify potential solutions to problems that occurred throughout the process of creating the CPGs. The FGDs were directed by a [name deleted to maintain the integrity of the review process] team member, and the participants included both [name deleted to maintain the integrity of the review process] employees and the Department of ENT. Prior to the FGDs, participants were given the chance to individually communicate the challenges they faced and propose solutions. Each obstacle was characterized as either a major or minor difficulty by agreement. The [name deleted to maintain the integrity of the review process] team then grouped the final list of individual challenges into broad themes with their suitable solutions.

### Development of Referral Pathways

The recommendations in our local CPG were used as the core material for the development of primary care management and referral pathways. The ENT specialists at [name deleted to maintain the integrity of the review process] worked with the [name deleted to maintain the integrity of the review process] staff to develop the management algorithms for primary care physicians. They focused on early diagnosis, management at the level of primary care, and referral to specialists when needed.

If any gaps in care provision were found while drafting the clinical management and referral pathways, we sought additional recommendations through a best-evidence systematic review process. This preferably involved the use of recommendations from already-existing CPGs other than the selected source CPGs. The evidence collected to develop the recommendation was then reviewed by the experts. If already-existing recommendations in other CPGs were not found, recommendations were drafted and included in the clinical management and referral pathways using peer-reviewed evidence from trustworthy information sources. If the best-evidence systematic review process resulted in no citable evidence, we made additions based on expert consensus.

## Results

We created local CPGs for the eight most common ENT disorders seen in the primary care setting of Pakistan using the GRADE-ADOLOPMENT method. Across the 108 recommendations found in the source CPGs for the 8 ENT disorders, 5 recommendations were adopted with minor changes into the local CPG (1 recommendation for AOE, SSNHL, and epistaxis, and 2 for AR), and 6 were excluded from the local CPGs (1 recommendation for epistaxis, 2 for SSNHL, and 3 for AR) ( Tables 1 2 ). All other recommendations were adopted as they were in our local CPGs. The finalized local CPGs can be found in **Additional File 1** .

**Table 1:** Recommendations adopted with minor changes in the CPGs

	Recommendation	Change made
**Acute otitis externa**		
1	“When a patient has a known or suspected perforation of the tympanic membrane, including a tympanostomy tube, prescribe a non-ototoxic topical preparation **(e.g., ciprofloxacin ear drops)** . [Recommendation, grade: D, level of confidence in evidence: Moderate]”	Description **(e.g., ciprofloxacin ear drops)** added. Ciprofloxacin is available as non-ototoxic ear drops.
**Epistaxis**		
1	“Nasal packing: **a)** For patients in whom bleeding precludes identification of a bleeding site despite nasal compression, the clinician should treat ongoing active bleeding with nasal packing **(with either merocele or cotton ribbon as available).** Nasal packing in patients with suspected bleeding risk – **b)** The clinician should use resorbable packing **(e.g., Gelfoam)** for patients with a suspected bleeding disorder or for patients who are using anticoagulation or antiplatelet medications.” [Recommendation, grade: C]	Description **(with either merocele or cotton ribbon as available)** added. Merocele and cotton ribbon are non-absorbable packing. Description **(e.g., Gelfoam)** added. Gel foam is a resorbable packing.
**Allergic Rhinitis**		
1	“Clinicians should recommend intranasal steroids ( **e.g., mometasone, fluticasone, beclomethasone)** for patients with a clinical diagnosis of AR whose symptoms affect their quality of life. [Grade: A, high level of confidence in evidence]”	Description ( **e.g., mometasone, fluticasone, beclomethasone)** added. Mometasone, fluticasone and beclomethasone are widely-available as intranasal preparations.
2	“Clinicians should recommend oral second-generation/less sedating antihistamines **(e.g., fexofenadine, cetirizine, loratadine)** for patients with AR and primary complaints of sneezing and itching. [Grade: A, high level of confidence in evidence]”	Description **(e.g., fexofenadine, cetirizine, loratadine)** added. Fexofenadine, cetirizine and loratadine are widely-available oral second-generation/less sedating antihistamines.
**Sudden Sensorineural Hearing loss**		
1	“In patients with SHL clinicians should obtain, or refer to a clinician who can obtain, audiometry **(pure tone audiometry and speech discrimination score)** as soon as possible (within 14 days of symptom onset) to confirm the diagnosis of SSNHL. [Recommendation, Grade: C]”	Description **(pure tone audiometry and speech discrimination score)** added. Both tests are required for an accurate assessment.

**Abbreviations:** AR, allergic rhinitis; CPGs, clinical practice guidelines; SHL, sudden hearing loss; SSNHL, sudden sensorineural hearing loss.

**Table 2:** Recommendations excluded from the source guideline for the management of ENT conditions in Pakistan

Action	Recommendation	Reason for exclusion
**Epistaxis**		
**Excluded**	**“Nosebleed Outcomes:** ** Outcome of intervention within 30 days or transition of care in patients who had a nosebleed treated with non-resorbable packing, surgery, or arterial ligation/embolization should be documented.” 23**	**Due to cost-effectiveness**
**Sudden Sensorineural Hearing loss**		
**Excluded**	**“Initial Therapy with Hyperbaric Oxygen Therapy:** ** HBOT combined with steroid therapy within 2 weeks of onset of SSNHL.” 26**	**Due to mass unavailability of HBOT**
**Excluded**	**“Salvage Therapy with Hyperbaric Oxygen Therapy:** ** HBOT combined with steroid therapy as salvage within 1 month of onset of SSNHL.” 26**	**Due to mass unavailability of HBOT**
**Allergic Rhinitis**		
**Excluded**	**“Inferior Turbinate Reduction:** ** Clinicians may offer, or refer to a surgeon who can offer, inferior turbinate reduction in patients with AR with nasal airway obstruction and enlarged inferior turbinate who have failed medical management.” 24**	**Due to cost-effectiveness**
**Excluded**	**“Acupuncture:** ** Offer acupuncture for patients with AR who are interested in nonpharmacologic therapy.” 24**	**Due to unavailability**
**Excluded**	**“Herbal Therapy:** ** No recommendation regarding the use of herbal therapy for patients with AR.” 24**	**Redundant recommendation**

**Abbreviations:** AR, allergic rhinitis; ENT, ear, nose, and throat; HBOT, hyperbaric oxygen therapy; SSNHL, sudden sensorineural hearing loss.

We also created primary care clinical-referral algorithms for these conditions based on the recommendations in the local CPGs ( **Additional File 2** ). We added four recommendations to the epistaxis algorithm, three to the neck lumps/masses, rhinosinusitis, and AR algorithm, two for the AOE algorithm, and one for the Ménière's disease and dysphonia algorithms ( Table 3 ).

**Table 3:** Recommendations added to the clinical-referral pathways

Added Recommendation		Source
**Epistaxis**		
1	Look for signs and symptoms – active nasal bleeding, spitting, or vomiting blood, dizziness, confusion, weakness, tachycardia, syncope, orthostatic hypotension, and amount of blood loss.	AAFP 37 38 AFP 39
2	Assess risk factors like nasal or facial trauma, prior nasal surgery, continuous positive airway pressure use, chronic kidney, or liver disease.	AAFP 38
3	Educate the patient regarding risk factors which can cause epistaxis, such as hot and dry environment, trauma, strenuous activity, digital trauma, excessive nose blowing and inappropriate drug use.	Medscape 40
4	Investigations such as complete blood count, prothrombin time, and partial thromboplastin time may be conducted in patients with suspected bleeding disorder.	AAFP 38
**Neck Masses/Lumps**		
1	Look for signs and symptoms – duration, change in size, hoarseness, dysphagia, sore throat, unexplained weight loss, dyspnea, and odynophagia.	AJGP 41
2	Assess risk factors, smoking, alcohol use, history of head and neck malignancy, inciting incident, family history, addictions, personal history of TB or exposure to TB patient, medication use and comorbid conditions.	AJGP 41
3	Suggest TB work-up if clinical suspicion of TB is present.	AAFP 42
**Acute Otitis Externa**		
1	Look for signs and symptoms – severe otalgia, itching, aural fullness, tenderness of the tragus, hearing loss, jaw pain, and otorrhea.	AAFP 43
2	Educate the patient regarding risk factors which can cause AOE such as moisture, water contaminated with bacteria, insertion of foreign objects in ear and chronic dermatological conditions.	AAFP 43
**Rhinosinusitis**		
1	Obtain comprehensive history and perform physical examination in patients to evaluate nasal or postnasal discharge, nasal obstruction, facial pain, pressure/fullness, fever, and hyposmia/anosmia.	AAFP 44
2	Offer oral steroids with proton pump inhibitors for nasal polyps in a patient with chronic rhinosinusitis (ensure adjusted dose for diabetic patients).	AAAAI 45 JBDS 46
3	Educate the patient regarding antibiotic use.	CDC 47
**Allergic Rhinitis**		
1	Clinicians should assess patients with a clinical diagnosis of AR for, and document in the medical record, the presence of associated conditions such as cystic fibrosis.	CFF 48
2	Recommend nasal saline irrigation for patients with allergic rhinitis.	BSACI 49
3	A complete blood count to see a raised eosinophil count for patients with a clinical diagnosis of allergic rhinitis may be considered.	Medscape 50
**Ménière's Disease**		
1	Educate patients with Ménière's disease about dietary and lifestyle modifications that may reduce or prevent symptoms, such as decreasing sodium and caffeine intake.	Medscape 51
**Dysphonia**		
1	Advise voice rest.	AFP 52

**Abbreviations:** AAFP, American Academy of Family Physicians; AAAAI, American Academy of Allergy, Asthma & Immunology; AFP, Australian Family Physician; AJGP, *Australian Journal of General Practice* ; AOE, acute otitis externa; AR, allergic rhinitis; BSACI, British Society of Allergy and Clinical Immunology; CDC, Centers for Disease Control and Prevention; JBDS, Joint British Diabetes Societies; TB, tuberculosis.

The challenges faced while developing the CPGs were grouped into three main themes: stakeholder support and involvement, resources, and resistance to change ( **Additional File 1** ).

## Discussion

The local CPGs we developed can guide GPs regarding the eight most common ENT-related patient complaints in the primary care setting. These CPGs provide patient management details, starting with a thorough clinical evaluation, including history, examinations, and investigations, followed by step-by-step management encompassing pharmacological and non-pharmacological modalities. These local CPGs were organized into comprehensive yet simple primary care management and referral pathways for GPs. These referral pathways were developed to make the recommendations in the CPGs more convenient for GPs to use and, when necessary, guide the decision for appropriate referrals to ENT specialists.

In the primary care setting, GPs function as gatekeepers to specialized ENT care. In fulfilling this role, GPs must be equipped with the knowledge of current evidence-based practices as applicable to their local setting, and they must be able to understand the extent of their role as care providers and determine when a patient warrants a referral to a specialist. This enables GPs to alleviate some of the burdens on the scarce specialist ENT resources while lowering healthcare costs, a consideration of particular importance in an LMIC setting. 4 28 Our CPGs and referral algorithms enable GPs to provide better health care at the patient's initial point of contact while bridging the gap between primary and specialized secondary healthcare. 29

Referral rates from GPs to specialized ENT services in the literature 30 31 32 are generally variable, ranging from 4.3 up to 20% of all patients seen by GPs with ENT complaints. This variability is attributable to differences in practices at the levels of the individual GP and the healthcare system. However, this variability also highlights the burden of potentially-inappropriate referrals to specialist ENT services, which, in an LMIC such as Pakistan, can further exacerbate shortages of specialist resources. Another form of inappropriate use of resources involves diagnostic tests. While baseline investigations are generally necessary to establish the correct diagnoses, they can be overused, causing a needless financial burden on the patients without significantly improving their health status. 33 Our newly-created local CPGs and clinical-referral pathways can help in the standardization of ENT care provision at the primary care level and help minimize unnecessary consumption of specialist resources.

In addition, the variability in certain management practices, such as the prescription of antibiotics for suspected infections, is also a matter of concern for antimicrobial stewardship efforts in the country. 34 Studies 35 36 have shown that medical doctors in Pakistan generally have some misconceptions regarding appropriate antibiotic prescriptions. Additionally, GPs often feel pressured to administer antibiotics even if they are not convinced that they were indicated, with a major proportion of them doing so solely on the basis of the patient's insistence. Thus, the element of patient education incorporated into our clinical-care pathways is of particular importance in Pakistan, where lower literacy rates mean that patients are unaware of the far-reaching harmful consequences of inappropriate antibiotic prescription. Therefore, the use of these latest CPGs can help curb inappropriate prescription practices that lead to antimicrobial resistance and improve the standard of care for patients.

## Strengths and Weaknesses

The current study has certain limitations. As the TOR review is performed by individual experts, it is a subjective process that may introduce biases in the decision to adapt, adopt, or de novo develop recommendations. Another limitation of the present study is that the experts who developed the guidelines were from the same tertiary hospital. A more diversified group of experts could reduce the likelihood of institutional bias. Additionally, the feasibility of the implementation of these recommendations in rural settings remains a concern. The rural areas of Pakistan lack the infrastructure needed to provide specialist services, should patients require them. Hence, financial and geographical barriers are significant challenges to the implementation of the clinical-referral pathways.

The strength of our CPG lies in the transparent and rigorous methodology used to create it. The transparency of the GRADE-ADOLOPMENT approach will encourage GPs to trust in the recommendations provided and make informed decisions accordingly. The detailed step-by-step referral pathways will help streamline the entire process and assist in patient triage. Our process for the development of CPGs and referral pathways may serve as a template for other resource-challenged countries, such as those in Africa, which experience similar challenges, 4 and other countries with similar population demographics and disease burdens to develop their own clinical guidelines and management algorithms.

## Conclusion

We used the GRADE-ADOLOPMENT approach to create eight local CPGs for common ENT disorders in Pakistan at the primary healthcare level. The recommendations provided will help in the provision of standardized, high-quality care by GPs across the country. Concomitantly, eight referral pathways were also developed to guide stepwise evaluation, management, and specialist referral of patients presenting with ENT disorders. We believe that these newly-created CPGs and referral pathways will enable the healthcare system in Pakistan to deliver the best possible standardized patient care for common ENT-related conditions.

## Notes

Additional File 1.pdf 1. Adapted GRADE-ADOLOPMENT approach. 2. GRADE-ADOLOPMENT approach for adaptation using evidence-to-decision (EtD) tables. 3. Supplementary Table 1: Criteria evaluated in the evidence-to-decision (EtD) tables. 4. Supplementary Table Section 2: Dummy evidence-to-decision (EtD) table. 5. Supplementary Table 3: Challenges faced while developing CPGs and proposed solutions. 6. Key to understanding level of evidence and strength of recommendation. 7. Table of Recommendations: Management of Acute Otitis Externa 8. Table of Recommendations: Management of Dysphonia 9. Table of Recommendations: Management of Epistaxis 10. Table of Recommendations: Management of Allergic Rhinitis 11. Table of Recommendations: Management of Neck Masses/Neck Lumps 12. Table of Recommendations: Management of Sudden Sensorineural Hearing loss 13. Table of Recommendations: Management of Sinusitis 14. Table of Recommendations: Management of Ménière's Disease

## Notes

Additional File 2.pdf **1.** Supplementary Fig. 1: Primary Care Referral Pathways for the Management of Epistaxis
**2.** Supplementary Fig. 2: Primary Care Referral Pathways for the Management of Neck Masses/Lumps
**3.** Supplementary Fig. 3: Primary Care Referral Pathways for the Management of Acute Otitis Externa
**4.** Supplementary Fig. 4: Primary Care Referral Pathways for the Management of Sudden Sensorineural Hearing Loss
**5.** Supplementary Fig. 5: Primary Care Referral Pathways for the Management of Rhinosinusitis
**6.** Supplementary Fig. 6: Primary Care Referral Pathways for the Management of Allergic Rhinitis
**7.** Supplementary Fig. 7: Primary Care Referral Pathways for the Management of Ménière's Disease
**8.** Supplementary Fig. 8: Primary Care Referral Pathways for the Management of Dysphonia
